# Early neuro biomarker changes and electrophysiological nerve dysfunction in Swedish V50M hereditary transthyretin amyloidosis: a pilot study

**DOI:** 10.3389/fneur.2026.1943011

**Published:** 2026-10-02

**Authors:** Sandra Arvidsson, Intissar Anan, Malin Olsson, Victoria Heldestad

**Affiliations:** 1 Department of Diagnostics and Intervention, Umea University, Umeå, Sweden; 2 Department of Public Health and Clinical Medicine, Umea University, Umeå, Sweden; 3 Department of Clinical Microbiology, Umea University, Umeå, Sweden; 4 Department of Clinical Science, Umea University, Umeå, Sweden

**Keywords:** ATTRv, nerve conduction studies (NCS), nerve ultrasound, neurofilament, peripherin (PRPH)

## Abstract

**Background:**

This exploratory pilot study aimed to evaluate serum levels of neurofilament light chain (NfL), peripherin (PRPH), and glial fibrillary acidic protein (GFAP), and to explore their potential associations with nerve conduction studies (NCS) and nerve ultrasound (NUS) in a Swedish cohort of newly diagnosed patients with V50M transthyretin (TTR) amyloidosis (ATTRv).

**Materials and methods:**

Seventeen V50M-ATTRv patients exhibiting varying initial clinical symptoms were included for NCS, NUS, and blood analyses of NfL, PRPH, and GFAP.

**Results:**

V50M-ATTRv patients had a short disease duration (median, 7 months) and predominantly (52.9%) presented with self-reported neuropathic clinical symptoms at disease onset. Sixteen had recently initiated TTR-stabilizing therapy, whereas one remained untreated. Overall, NfL levels were significantly higher in patients than in age-matched controls (*p* < 0.001). This difference remained significant among older individuals (*p* < 0.001), but not among younger individuals (*p* = 0.071). Patients with an earlier age at disease onset had significantly higher PRPH levels compared with those with later disease onset (*p* = 0.030). Among younger individuals, PRPH levels were higher in patients compared with age-matched controls, but the difference was not significant (*p* = 0.200). NCS parameters showed significant associations with both NfL and PRPH, but no such associations were seen with GFAP. None of the neurobiomarkers demonstrated any associations with NUS.

**Conclusion:**

In this pilot cohort of newly diagnosed Swedish V50M-ATTR patients, NfL was associated with electrophysiological measures of axonal dysfunction, whereas PRPH showed an exploratory signal of interest in younger patients with early disease onset. Larger longitudinal studies are required to determine whether PRPH may serve as a complementary biomarker for the early detection and monitoring of peripheral nerve involvement in ATTRv. Given the pilot design and limited sample size, the subgroup and regression findings should be considered exploratory and hypothesis-generating.

## 1 Introduction

Hereditary transthyretin amyloidosis (ATTRv) is a rare, progressive, and ultimately fatal disease with over 200 different point mutations identified worldwide (1). In European populations, particularly in Sweden, the p. Val50Met (V50M) variant, formerly designated V30M, is most frequently observed (2, 3). Misfolded transthyretin (TTR) leads to the formation of amyloid fibrils, which predominantly deposit in the heart, eyes, and peripheral nerves (4). Two distinct fibrillar forms, type A and B, have been identified in V50M-ATTRv. Type A fibrils comprise a mixture of fragmented and full-length TTR and are more common in individuals with a later age at disease onset. Early-onset patients (<50 years of age) appear to predominantly exhibit type B fibrils, which consist of full-length TTR, and have a higher tendency to develop neuropathic symptoms (5). Although no curative treatment is currently available, several drugs have been shown to slow disease progression (6–9), emphasizing the importance of early diagnosis and timely initiation of treatment. Variability in clinical manifestation, differences in penetrance between endemic areas (2, 4), and delayed diagnosis in non-endemic areas (10) further underscore the continued need for more advanced diagnostic methods.

Among potential neurobiomarkers, neurofilament light chain (NfL) is considered one of the most promising, as it reflects axonal degeneration across a wide range of neurological diseases affecting the central (CNS) and/or peripheral nervous system (PNS) (11–14). Glial fibrillary acidic protein (GFAP), an intermediate filament protein, is primarily regarded as a marker of central nervous system injury (15). However, GFAP has also shown potential as a predictor of axonal damage and clinical outcomes in acute peripheral neuropathies, such as Guillain–Barré syndrome (GBS) (16). Peripherin (PRPH), by contrast, is more specifically associated with peripheral axonal degeneration (17). Axonal loss in the V50M-ATTR mutation is primarily attributed to the degeneration of small nerve fibers (4, 18–20). Consequently, progressive disability in nerve conduction studies (NCS) showing reduced or absent sensory nerve action potentials (SNAPs) and compound muscle action potentials (CMAPs) (19, 21). In contrast, nerve ultrasound (NUS) often demonstrates an enlarged cross-sectional area (CSA) in patients with ATTRv (22–24), similar to findings in inflammatory polyneuropathies (25, 26). However, there is still no established consensus regarding early NUS findings or their association with specific ATTRv point mutations.

If left untreated, ATTRv leads to death within 5–12 years after symptom onset (4, 19). Therefore, early diagnosis is crucial and requires the integration of blood biomarkers with comprehensive clinical examinations. Consequently, NfL is implied to have diagnostic and prognostic value in ATTRv, especially in patients with a predominant neuropathic phenotype (12, 13, 27, 28), while recent evidence highlights PRPH as a promising biomarker in a cohort with mixed genotypes and phenotypes (29). However, the majority of previous biomarker studies in ATTRv have relied on genetically and clinically heterogeneous cohorts, often with variable disease durations and treatment statuses. Less is known about how circulating neurobiomarkers relate to objective electrophysiological and sonographic nerve parameters early after disease onset in a genetically homogeneous V50M cohort.

Therefore, this study aimed to evaluate serum NfL, PRPH, and GFAP in newly diagnosed Swedish V50M-ATTRv patients, and to explore their associations with electrophysiological and sonographic nerve parameters, along with age at disease onset, initial clinical phenotype, and fibril type.

## 2 Materials and methods

### 2.1 Patients

This pilot study was a cross-sectional, single-center study conducted between 2021 and 2024 at Umeå University. Clinical data were collected, and blood sampling was performed at the national specialist center for ATTRv amyloidosis at Umeå University Hospital, Sweden. Patients with confirmed a ATTRv diagnosis were included, which was established according to national clinical routines and requirements: (1) a positive TTR gene test, and (2) evidence of amyloid deposits in a fat pad biopsy and/or positive uptake on cardiac 3,3-diphosphono-1,2-propanodicarboxylic acid (DPD) scintigraphy.

Initial clinical manifestations reported by patients were categorized into the following distinct phenotypes: neuropathic, cardiac, gastric, ocular, and renal. Patients with features of two or more phenotypes were classified as having a mixed phenotype. Medical records were reviewed to obtain information regarding patient creatinine levels, eGFR, disease duration, and treatment initiation. Estimation of disease duration was based on the time interval between confirmed ATTRv diagnosis and blood sampling, NCS, or NUS. All patients underwent blood sampling for neurobiomarker analysis and clinical examinations using NCS and NUS. Because treatment had been initiated less than 30 days before biomarker sampling and neurophysiological assessment in 16 of the 17 patients, treatment effects on the measured biomarkers were considered unlikely but could not be fully excluded.

### 2.2 Biomarkers control cohort

Twenty-seven matched controls were included for neurobiomarker analysis. All were genotyped to confirm the absence of the V50M variant (p. Val50Met), and none of the controls had a reported clinical history of neuropathy or cardiac symptoms (i.e., they were subjectively healthy).

### 2.3 Serum NfL, GFAP and PRPH measurements

Serum concentrations of NfL, GFAP, and PRPH were measured using enzyme-linked immunosorbent assays (ELISAs). Blood samples were collected in serum tubes without gel separators, allowed to clot at room temperature, and centrifuged at 1.600 × *g* for 16 min. The resulting serum was aliquoted and stored at −80 °C until analysis. Serum NfL levels were quantified using a commercial ELISA kit (Cat. No. 20–8,002; UmanDiagnostics AB, Umeå, Sweden). GFAP concentrations were measured with the Invitrogen ELISA kit (Cat. No. EEL079; Thermo Fisher Scientific Inc., Waltham, MA, USA), and PRPH concentrations were determined using a PRPH-specific ELISA kit (Cat. No. BSKH64124; Bioss Inc., Woburn, Massachusetts, USA). Serum samples were analyzed in duplicate following the manufacturer’s instructions for all assays. Briefly, blanks, standards, and appropriately diluted serum samples were pipetted into precoated 96-well microplates and incubated at the recommended temperatures for each assay. After washing, conjugated detection antibodies were added and incubated, followed by the addition of the substrate solution. The reaction was terminated with an acid-stop solution, and absorbance was measured at 450 nm using a SkyHigh microplate reader (Thermo Scientific Multiskan, USA). Serum biomarker concentrations were then automatically calculated using SkanIt software (Thermo Scientific, USA).

### 2.4 Nerve conduction studies

Nerve conduction studies were performed with a Nicolet Viking Select system (Nicolet Biomedical®, Madison, USA); the filter range was 20 Hz to 1.5 kHz, the sweep duration was 1 ms/division, the stimulation frequency was 2 Hz, and the pulse duration was 0.2 ms. Electrical stimulations were conducted at conventional sites for the fibular and tibial nerves (the ankle and knee). Compound muscle action potentials were recorded at the abductor hallucis and extensor digitorum brevis using 10-mm surface disk electrodes (Alpine Biomed Hush™, Skovlunde, Denmark), with a fixed distance of 80 mm between the distal stimulation site and the active surface disk electrode. Sensory NCS were performed on the sural nerves with an antidromic technique, using the same surface disk electrodes for SNAP recordings. The active electrode was positioned posterior to the lateral malleolus, with a distance of 140 mm between the stimulation and recording sites at the ankle. Motor and sensory nerve amplitudes were measured from baseline to peak. All motor and sensory NCS were performed bilaterally, and to ensure compliance with local laboratory standards, the dorsal foot skin temperature was continuously monitored and maintained above 30 °C throughout the procedure.

### 2.5 Nerve ultrasound

Nerve ultrasound was performed by an experienced sonographer, using the same device throughout the study: a Canon Aplio A450 (© Canon Medical Systems Europe B.V., 2020), equipped with an 18 L7 (12–18 MHz) transducer, model PLT-1204BT (© Canon Medical Systems Europe B.V., 2020). The sural, tibial, and fibular nerves were examined bilaterally in the short-axis view, according to previously described anatomical positions (22). Depth and focus settings were adjusted according to the nerve’s anatomical location, and the most frequently used depth setting was 2–3 cm to optimize imaging of the epineurium. The CSA for each nerve site was measured by manually tracking the inner epineurium and calculated as the mean of the two highest-quality ultrasound images.

### 2.6 Statistical analysis

Statistical analyses were performed with Excel (Microsoft, Redmond, WA, USA) and SPSS (IBM, Armonk, NY, USA). Descriptive data, including CMAP (mV), CSA (mm^2^), SNAP (μV), creatinine [μmol/L], eGFR [mL/min/1.73 m^2^], NfL, PRPH, and GFAP [pg/mL], were described as medians and ranges (minimum–maximum) or 25th–75th percentiles (interquartile range, IQR). The Mann–Whitney U test and Kruskal–Wallis test were used to test for differences between groups. Correlation analyses were performed using Spearman’s correlation coefficient and were classified as strong (0.70–1.00) or moderate (0.40–0.69) (30). Linear multiple regressions were considered exploratory and hypothesis-generating and were performed with serum neurobiomarkers as dependent variables, using age and BMI as predictors, and with clinical nerve examination parameters as dependent variables, using NfL, PRPH, and GFAP as predictors. The one-sample Kolmogorov–Smirnov test was used to assess the normality of PRPH levels before and after log10 transformation. A *p*-value of *<*0.05 was considered statistically significant.

### 2.7 Ethics

All participants provided oral and written informed consent. The study followed the ethical guidelines of the Declaration of Helsinki and was approved by the Swedish Ethics Review Authority (2021/03826, 2022/04551, 2024–01465-02).

## 3 Results

### 3.1 Demographics

Seventeen V50M-ATTRv patients (13 male patients), aged between 30 and 87 years (median, 68 years), were included in the study. The majority of them (88%) had late-onset disease (clinical diagnosis at ≥50 years of age), with a median (range) disease duration of 7 (2–16) months. Treatment with TTR stabilizers was recently initiated in 16 patients, i.e., <30 days before blood sampling and neurophysiological testing (i.e., NCS and NUS). One patient had no ongoing treatment. Eleven (65%) were fibril-typed. The proportions of initial clinical symptoms were exclusively neuropathic (i.e., numbness and tingling in the feet) in 52.9%, cardiac (17.6%), renal (5.9%), and ocular (5.9%), or mixed (17.6%). No patients reported gastric symptoms alone (constipation and/or obstipation) as the initial clinical manifestation of the disease. All three patients with mixed symptom presentations reported numbness and tingling in the feet, in combination with constipation and/or obstipation; additionally, one reported dyspnea. None of the patients had a diagnosis of carpal tunnel syndrome (CTS). Descriptive data are shown in Table 1.

**Table 1:** Descriptive data, disease duration, blood samples, fibril types and initial clinical symptoms in 17 patients with hereditary transthyretin amyloidosis (ATTRv).

Data	Median (IQR)	*p*-value
Age (years)	68 (58–73)	n.s
Height (cm)	178 (166–182)	
Weight (kg)	78.2 (65.9–91.8)	
Body Mass Index (kg/m^2^)	25.8 (22.4–28.1)	
Disease duration (months)	7.0 (3.0–8.0)	
Glial fibrillary acidic protein [pg/mL]	640.0 (520.5–858.3)	n.s
Neurofilament light chain [pg/mL]	59.4 (30.3–94.4)	<0.001
Peripherin [pg/mL]	149.2 (22.5–1410.4)	n.s
Creatinine [μmol/L]	74.5 (70.2–89.8)	
eGFR [mL/min/1.73 m^2^]	89.0 (72.3–89.8)	

Data	Number of subjects
Males/females	13/4
Late/early disease onset	15/2
Fibril type A/B	6/5
Initial clinical symptoms	
Neuropathic	9
Cardiac	3
Visual	1
RenaL	1
Mixed	3

eGFR, estimated glomerular filtration rate; n.s, not significant. *p*-values represent comparisons between ATTRv patients and matched controls.

The 27 included matched controls for blood marker analysis (16 males) were aged between 31 and 88 years (median, 60.9 years). They were stratified into two similar age groups according to the age groups used for ATTRv patients: younger (<50 years of age) and older (≥50 years of age) (see Table 2). There were no significant differences in age between matched controls and V50M-ATTRv patients (*p* = 0.264).

**Table 2:** Descriptive data and neuro biomarkers concentrations in 27 matched controls.

Data	Median (IQR)
Age (years)	60.9 (60.7–74.1)
Glial fibrillary acidic protein^a^ [pg/mL]	595.6 (473.7–761.7)
Neurofilament light chain^b^ [pg/mL]	15.23 (10.3–23.8)
Peripherin^c^ [pg/mL]	451.2 (11.3–1253.0)

Data	Number of subjects
Males/Females	16/11
Older (≥50 years)/younger (<50 years)	21/6

a Missing data in one subject.

b Missing data in five subjects.

c Missing data in 13 subjects.

### 3.2 Blood markers

#### 3.2.1 Neuro biomarkers and renal blood markers in V50M-ATTRv

In total, serum levels of GFAP ranged between 380.7 and 1,057.0 pg./mL, levels of NfL between 12.6 and 167 pg./mL, and levels of PRPH between 16.7 and 6,296 pg./mL. PRPH serum concentrations were right-skewed, with a few high levels among younger individuals (*p* = 0.005; *p* = 0.200 after log10 transformation). As shown in Figures 1A,B, serum concentrations of GFAP and NfL differed by age at disease onset, but not significantly. However, higher serum PRPH levels were seen in patients with early-onset disease compared with later-onset disease (Figures 1C,D). Regarding differences according to patients’ initial symptoms, serum levels of GFAP differed significantly between patients with neuropathic, cardiac, or mixed symptoms (Figure 2A). Visually, patients with neuropathic symptoms demonstrated higher serum levels of PRPH, but neither PRPH nor NfL levels differed significantly between symptom groups (Figures 2B,C). There were no significant differences in disease duration between these three subgroups (*p* = 0.523), and none of the neurobiomarkers demonstrated any significant differences between fibril subtypes (NfL, *p* = 0.429; GFAP, *p* = 0.792; PRPH, *p* = 0.662).

**Figure 1:**
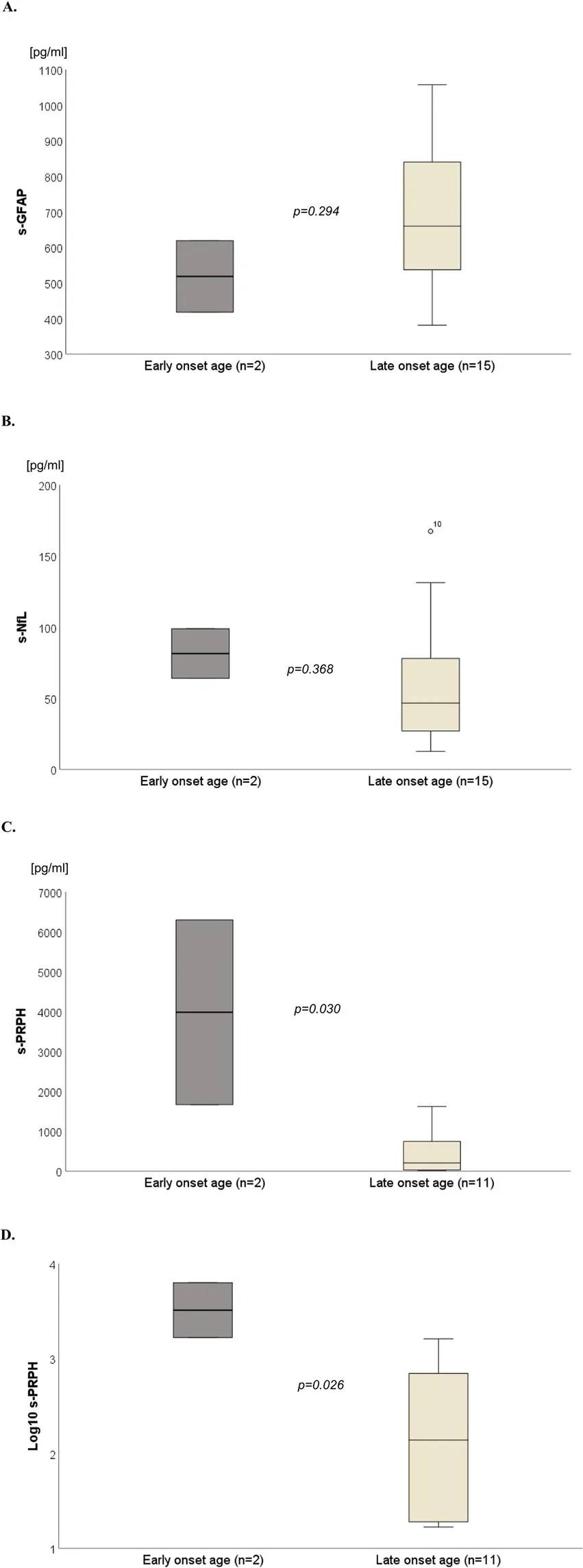
Boxplots showing the analyzed serum concentrations of **(A)** the glial fibrillary acidic protein (s-GFAP), **(B)** the neurofilament light chain (s-NfL), **(C)** the peripherin (s-PRPH), and **(D)** the log10 transformation of peripherin (logPRPH) in patients with hereditary transthyretin amyloidosis (ATTRv), divided between patients with early (< 50 years of age), and late disease onset age (≥ 50 years of age). The box shows median and percentiles between 25–75. The whiskers show the 95-percentage confidence interval (dots, values outside the confidence interval).

**Figure 2:**
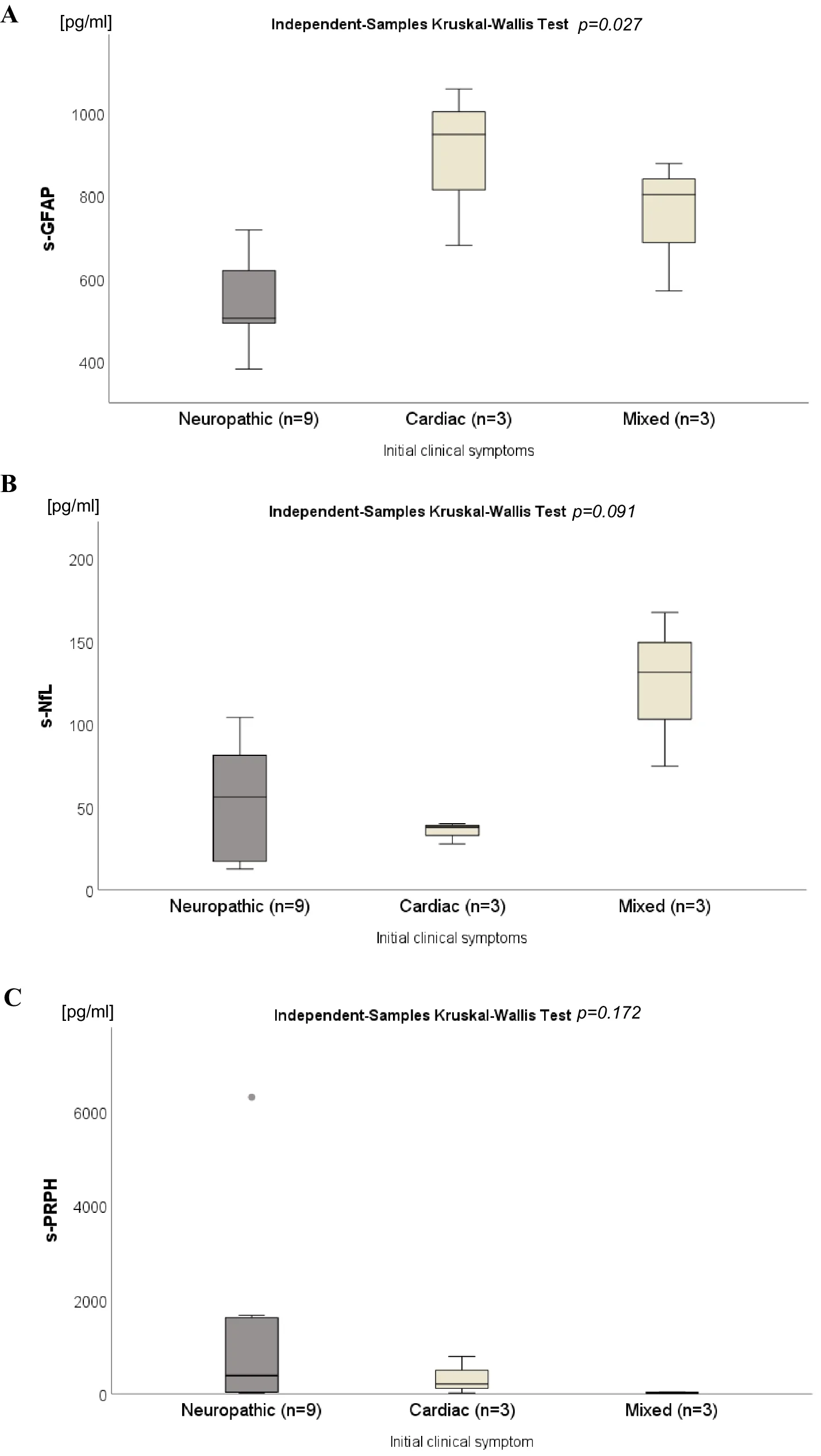
Boxplots showing analyzed serum concentrations of **(A)** the glial fibrillary acidic protein (s-GFAP), **(B)** the peripherin (s-PRPH), **(C)** the neurofilament light chain (s-NfL) in patients with hereditary transthyretin amyloidosis (ATTRv), divided between patients having exclusively neuropathic symptoms (n=9), cardiac (*n*=3) and mixed (*n*=3) symptoms. The box shows median and percentiles between 25–75. The whiskers show the 95-percentage confidence interval (dots, values outside the confidence interval).

Multiple linear regression analyses were conducted to assess the effects of age and BMI on serum levels of neurobiomarkers. A significant overall model was identified for GFAP, explaining 69.9% of the variance (*R*^2^ = 0.699, *p* < 0.001). BMI emerged as a strong independent predictor (*β* = 0.759, *p* < 0.001), while age had no independent contribution (*β* = 0.265, *p* = 0.102). No overall significant model was found for NfL (*R*^2^ = 0.058, *p* = *0.*748); neither BMI (*β* = −0.074, *p* = 0.805) nor age (*β* = −0.236, *p* = 0.439) was a significant predictor. Similarly, the model for PRPH did not reach overall significance (*R*^2^ = 0.494, *p* = 0.071). However, age was identified as a significant independent predictor (*β* = −0.719, *p* = 0.026), while BMI was not associated with PRPH (*β* = 0.258, *p* = 0.981).

Renal blood parameters ranged from 61 to 104.0 μmol/L (creatinine) and from 58 to >90 mL/min/1.73 m^2^ (eGFR), but they did not differ according to age at onset or between patients with exclusively neuropathic symptoms and those with other symptoms. Moreover, no significant correlations were noted between renal blood parameters and age or BMI. Two patients exhibited elevated levels of creatinine, and one had decreased eGFR [i.e., hospital laboratory cut-off reference values: creatinine >90 μmol/L (female patients), >100 μmol/L (male patients), eGFR <80 mL/min/1.73m^2^ (subjects <50 years) and <60 mL/min/1.73 m^2^ (subjects ≥50 years)].

#### 3.2.2 Neuro biomarkers in matched controls

In age-matched controls, serum levels of GFAP ranged between 150.6 and 1,214.0 pg./mL (median, 595.6 pg./mL), levels of NfL between 7.6 and 62.6 pg./mL (median, 15.2 pg./mL), and levels of PRPH between 11.0 and 3,458.0 pg./mL (median, 451.2 pg./mL). Blood analyses were missing in some subjects due to values below the limit of detection or a coefficient of variability above 15% (see Table 2 for details).

#### 3.2.3 Differences between V50M-ATTRv and controls

At group level, serum NfL levels were significantly higher in patients than in controls (*p* < 0.001). Significantly higher NfL levels were also seen in patients compared with controls among older subjects (Figure 3A), but not among younger subjects, although a visual difference was observed. Regarding PRPH, a non-significant trend toward higher PRPH levels was observed in younger patients compared with age-matched controls (Figure 3B), whereas this pattern was not observed among older individuals. At the whole group-level, no significant difference between V50M-ATTRv patients and controls was seen for PRPH (*p* = 0484; *p* = 0.689 log10 transformation) or GFAP (*p* = 0.455).

**Figure 3:**
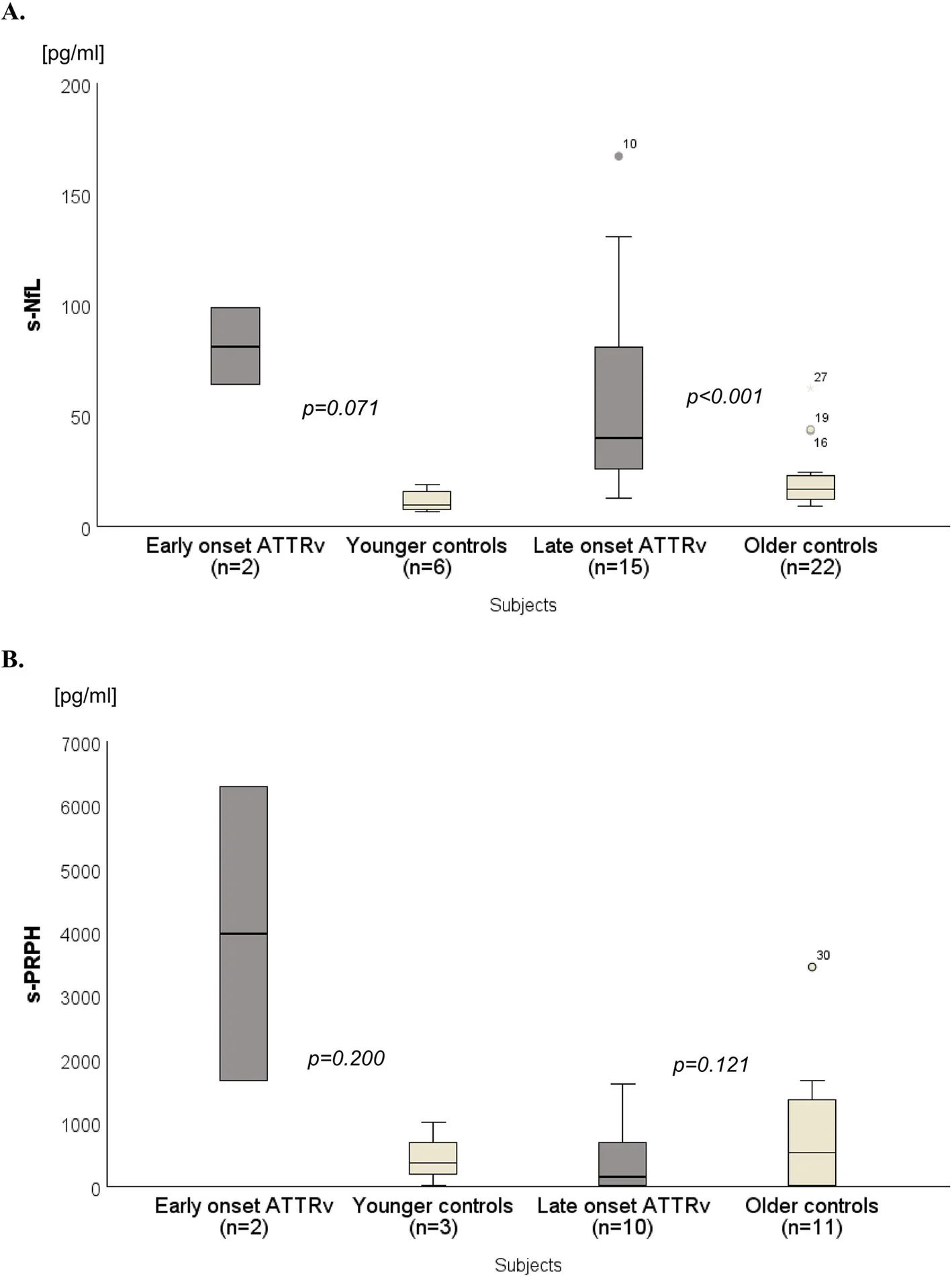
Boxplots showing differences between patients with hereditary transthyretin amyloidosis (ATTRv) and matched controls, divided between the patients and the controls < 50 years of age and ≥ 50 years of age. Serum levels of **(A)** neurofilament light chain (s-NfL) and **(B)** peripherin (s-PRPH). The box shows the median and percentiles between 25 and 75. The whiskers show the 95-percentage confidence interval (dots, values outside the confidence interval).

### 3.3 Clinical nerve parameters

Sural SNAPs showed a median (IQR) of 4.5 (2.5–7.2) μV. Compound muscle action potentials recorded following ankle stimulation at the fibular and tibial nerves demonstrated median (IQR) values of 2.7 (1.8–4.4) mV and 4.7 (1.0–10.3) mV, respectively. In addition, stimulation showed median (IQR) values of 2.5 (1.7–4.0) mV at the fibular nerve and 4.1 (0.8–7.4) mV at the tibial nerve. As no significant differences were found between sides, NCS data were pooled. Of the nine patients with exclusively neuropathic manifestations, five had SNAP and CMAP values within local hospital normal reference ranges, two of whom were classified as having early-onset disease.

Serum NfL demonstrated moderate to strong negative correlations with peripheral nerve conduction parameters, specifically sural SNAP (rho = −0.524, *p* = 0.045) and tibial CMAP recorded at both ankle (rho = −0.657, *p* = 0.008) and knee stimulation sites (rho = −0.647, *p* = 0.007). No significant correlations were observed between NCS and serum levels of PRPH or GFAP. Multiple linear regression analyses were subsequently performed to evaluate the predictive value of serum neurobiomarkers for clinical nerve parameters. For sural SNAP, the overall model explained 32.0% of the variance (*R*^2^ = 0.320), although the model did not reach overall statistical significance (*p* = 0.158). Nevertheless, serum NfL emerged as a significant independent predictor (*β* = −0.506, *p* = 0.046). For tibial CMAP recorded at ankle stimulation, the model explained 54.9% of the variance (*R*^2^ = 0.549, *p* = 0.013), and both serum NfL (*β* = −0.531, *p* = 0.014) and PRPH (*β* = 0.478, *p* = 0.025) were significant predictors. Similarly, tibial CMAP at knee stimulation demonstrated a significant model fit (*R*^2^ = 0.539, *p* = 0.015), where serum NfL was identified as a significant predictor (*β* = −0.564, *p* = 0.011), whereas PRPH did not reach significance (*β* = 0.403, *p* = 0.054). No significant associations were observed between serum neurobiomarker levels and fibular CMAP parameters or summarized CSA measures. Additionally, CSA at all three nerve sites was significantly larger in V50M ATTRv patients compared with a cohort derived from local hospital data (*p* < 0.001). Furthermore, none of the analyzed nerve conduction parameters differed significantly between the fibril subtypes.

## 4 Discussion

In this exploratory pilot study of newly diagnosed V50M-ATTRv patients, we demonstrated that serum levels of NfL, regardless of patients’ initial self-reported clinical symptoms, were significantly higher in patients compared with controls. This discrepancy was also observed among exclusively older individuals, which further reinforces the usefulness of evaluating NfL in patients with V50M-ATTRv. Although increasing age has been found to have an impact on NfL in healthy individuals (31), this can be accounted for by our finding of significantly higher levels of NfL in older V50M-ATTRv patients compared with older age-matched controls. Interestingly, serum levels of PRPH were significantly higher in patients with early-onset disease compared with those with late-onset disease. This finding should be considered preliminary and exploratory due to the small number of early-onset patients. However, this lower number was expected, as late-onset cases are more common in Sweden (32). Higher levels of serum PRPH were also found in younger V50M-ATTRv patients compared with age-matched controls. Although this observation did not reach significance, it may reflect limited statistical power resulting from the small sample size of early-onset patients. In addition, serum levels of NfL correlated significantly with several of the evaluated electrophysiological nerve parameters, and multiple regression analyses showed that electrophysiological nerve parameters, to some extent, were related to both NfL and PRPH. A limitation of the study was the lack of available assessments targeting small nerve fiber dysfunction, such as Sudoscan. This is noteworthy, as small nerve fiber impairment is considered an early pathological feature of V50M-ATTRv and may precede detectable large-fiber degeneration (20).

Our findings of markedly increased PRPH concentrations in younger patients compared with older patients could reflect more rapid neuropathic progression among younger individuals with V50M-ATTRv. This is noteworthy, as our two patients with early disease onset both had normal NCS and noticeably higher, although not statistically significant, PRPH levels compared with age-matched controls. Furthermore, previous studies have shown that higher levels of PRPH are associated with early impairment of sensory and motor neurons according to its crucial structural function (33, 34). This finding is of importance, as decreased SNAP and CMUP amplitudes are seen later in the disease course (21). Despite the small sample size in our cohort, we propose that this pilot study supports recent findings of PRPH as a promising biomarker in ATTRv (29), as our cohort had a notably shorter disease duration, had been treated for less than 1 month before blood sampling and clinical examinations, and had a homogeneous genotype.

To evaluate the extent to which neurobiomarkers predict clinical nerve parameters, we conducted a series of multiple regression analyses. However, due to our limited cohort size, these analyses should be seen as preliminary and hypothesis-generating. To minimize potential bias related to carpal tunnel syndrome (CTS), which is frequently observed in ATTRv (4, 19), we excluded NCS data from the upper extremities from our analysis, even though none of the patients were diagnosed with CTS, as the objective was to specifically assess early distal neuropathic axonal degeneration. Our analysis demonstrated that both NfL and PRPH functioned as significant independent predictors in our cohort of newly diagnosed patients, whereas GFAP did not. Previous studies have also shown promising results regarding the usefulness of sNfL as a diagnostic and prognostic biomarker in ATTRv (12, 13, 28, 35, 36). However, direct comparison is difficult, as endemic areas, study protocols, genotypes, phenotypes, inclusion or exclusion of patients receiving disease-modifying treatment, as well as the definition of disease diagnosis, differ. Furthermore, we did not observe any correlation between neurobiomarkers and sonographic measurements. Although we initially hypothesized that such associations might exist, given that elevated neurobiomarker levels have been reported in chronic inflammatory neuropathies and GBS (14, 16, 17) and that increased nerve CSA has been documented in inflammatory neuropathies as well as in ATTRv at various described nerve sites (22–26), this relationship did not emerge in our cohort. Recently, Camelo-Filho et al. (37) demonstrated nerve enlargement at upper-limb and proximal sites, including the median nerve, brachial plexus, and C6 root, with abnormalities also detectable in asymptomatic TTR variant carriers. These findings suggest that structural nerve alterations may occur at anatomical sites not examined in the present study, which focused on the sural, tibial, and fibular nerves. Consequently, the absence of associations between circulating neuro biomarkers and NUS measurements in our cohort does not exclude potential relationships with structural abnormalities at other, particularly proximal nerve sites. Nerve enlargement in ATTRv is, however, more heterogeneously distributed than in other neuropathies, and is thought to result from local amyloid deposition and impaired microcirculation, leading to interstitial edema and subsequent nerve swelling (38). Nevertheless, the precise mechanisms underlying these structural alterations remain unclear.

The remarkably high levels of GFAP in patients with a cardiac phenotype were somewhat surprising, given that these patients did not exhibit neurological manifestations of ATTR disease but could be related to older age. However, we did not identify any effects of age or BMI in the blood sample analyses, with the exception of GFAP levels, for which BMI emerged as a prominent contributing factor. Lower BMI has previously been associated with higher circulating GFAP concentrations (39), a relationship proposed to reflect dilution effects whereby individuals with higher BMI exhibit lower biomarker levels due to greater blood volume. Age, BMI, and renal function have also been shown to influence NfL concentrations in neuropathic diseases affecting both the central nervous system (40, 41) and the PNS (12, 42), as well as in healthy individuals (31). Thus, our findings do not rule out the possibility that serum NfL levels in patients with reduced eGFR should be interpreted with greater caution, and that both age and BMI may need to be considered in clinical assessments. We could not identify any differences between fibril types, either between patients with neuropathic, cardiac, or mixed symptoms, or among the different neurobiomarkers.

Our study has some limitations that need to be addressed. First, 35% of the patients could not be fibril-typed due to the small amount of amyloid. Second, the majority of patients had late-onset V50M-ATTRv and were more likely to exhibit fibril-type A (5). Therefore, more patients with early-onset disease would be favorable in future studies. Third, the limited sample size should be acknowledged, particularly for subgroup and regression analyses, which should therefore be interpreted as exploratory and hypothesis-generating. Another limitation was the presence of missing blood analysis data for a subset of individuals, particularly PRPH among controls. Furthermore, although 16 patients had recently initiated TTR-stabilizing therapy, treatment duration was less than 30 days before sampling and testing. A substantial treatment-related effect on biomarker levels is therefore unlikely, but this cannot be fully excluded in a cross-sectional design. However, treatment would likely have reduced biomarker levels, which in our study would have diminished group differences and thereby potentially strengthened our findings. Nevertheless, all patients shared a homogeneous genotype and exhibited a relatively short disease duration, which supports the biological plausibility of the findings.

## 5 Conclusion

In conclusion, this exploratory study suggests that serum NfL and PRPH may be useful biomarkers of early axonal involvement in Swedish V50M-ATTRv amyloidosis. NfL differentiated patients from age-matched controls and was associated with electrophysiological measures of nerve dysfunction. PRPH showed a signal of interest in younger and early-onset patients, but this observation requires confirmation in larger cohorts. These findings support further longitudinal studies evaluating NfL and PRPH as complementary biomarkers for early detection and monitoring of peripheral nerve involvement in ATTRv. Given the limited sample size and particularly the small number of early-onset patients, the observed PRPH findings should be considered exploratory and hypothesis-generating. Larger longitudinal cohorts, including presymptomatic carriers followed through disease conversion, will be required to determine whether PRPH provides complementary value for early detection and monitoring of peripheral nerve involvement in ATTRv.

## Data Availability

The datasets presented in this article are not readily available. Anonymized data may be available from the corresponding author upon reasonable request, subject to ethical and legal restrictions. Requests to access the datasets should be directed to victoria.heldestad@umu.se.
