# Correction: Early neuro biomarker changes and electrophysiological nerve dysfunction in Swedish V50M hereditary transthyretin amyloidosis: a pilot study

**DOI:** 10.3389/fneur.2026.2029529

**Published:** 2026-10-29

**Authors:** Sandra Arvidsson, Intissar Anan, Malin Olsson, Victoria Heldestad

**Affiliations:** 1 Department of Diagnostics and Intervention, Umea University, Umeå, Sweden; 2 Department of Public Health and Clinical Medicine, Umea University, Umeå, Sweden; 3 Department of Clinical Microbiology, Umea University, Umeå, Sweden; 4 Department of Clinical Science, Umea University, Umeå, Sweden

**Keywords:** ATTRv, nerve conduction studies (NCS), nerve ultrasound, neurofilament, peripherin (PRPH)

In the published article, there was a mistake in Table 2. Peripherin [pg/ml] should be Peripherin [pg/mL].

**Table 2:** Descriptive data and neuro biomarkers concentrations in 27 matched controls.

Data	Median (IQR)
Age (years)	60.9 (60.7–74.1)
Glial fibrillary acidic protein^a^ [pg/mL]	595.6 (473.7–761.7)
Neurofilament light chain^b^ [pg/mL]	15.23 (10.3–23.8)
Peripherin^c^ [pg/mL]	451.2 (11.3–1253.0)
Data	Number of subjects
Males/females	16/11
Older (≥50 years)/younger (< 50 years)	21/6

^a^Missing data in one subject.

^b^Missing data in five subjects.

^c^Missing data in 13 subjects.

The corrected Table 2 appears below.

Further, the reference for 11 was erroneously written as Ciardullo S, Muraca E, Bianconi E, Cannistraci R, Perra S, Zerbini F, et al. Diabetes mellitus is associated with higher serum Neurofilament light chain levels in the general US population. *J Clin Endocrinol Metab*. (2023) 108:361–7. doi: 10.1210/clinem/dgac580

It should be Gaetani L, Blennow K, Calabresi P, Di Filippo M, Parnetti L, Zetterberg H. Neurofilament light chain as a biomarker in neurological disorders. *J Neurol Neurosurg Psychiatry*. (2019) 90:870–81. doi: 10.1136/jnnp-2018-320106

The original version of this article has been updated.

